# Evaluation of fluence‐smoothing feature for three IMRT planning systems

**DOI:** 10.1120/jacmp.v11i2.3035

**Published:** 2010-04-16

**Authors:** Christopher J. Anker, Brian Wang, Matt Tobler, Julie Chapek, Dennis C. Shrieve, Ying J. Hitchcock, Bill J. Salter

**Affiliations:** ^1^ Department of Radiation Oncology. Huntsman Cancer Hospital University of Utah Salt Lake City UT USA 84112

**Keywords:** IMRT, smoothing, complexity, efficiency, conformality

## Abstract

Commercially available intensity‐modulated radiation therapy (IMRT) inverse treatment planning systems (ITPS) typically include a smoothing function which allows the user to vary the complexity of delivered beam fluence patterns. This study evaluated the behavior of three ITPSs when varying smoothing parameters. We evaluated four cases treated with IMRT in our clinic: sinonasal carcinoma (SNC), glioblastoma multiforme (GBM), base of tongue carcinoma (BOT), and prostate carcinoma (PST). Varian Eclipse v6.5, BrainLAB BrainScan v5.31, and Nomos Corvus v6.2 ITPSs were studied for the SNC, GBM, and PST sites. Only Eclipse and Corvus were studied for BOT due to field size constraints of the BrainLAB MM3 collimator. For each ITPS, plans were first optimized using vendor‐recommended default “smoothing” values. Treatment plans were then reoptimized, exploring various smoothing values. Key metrics recorded included a delivery complexity (DC) metric and the Ian Paddick Conformality Index (IPCI). Results varied widely by vendor with regard to the impact of smoothing on complexity and conformality. Plans run on the Corvus ITPS showed the logically anticipated increase in DC as smoothing was decreased, along with associated improved organ‐at‐risk (OAR) sparing. Both Eclipse and BrainScan experienced an expected trend for increased DC as smoothing was decreased. However, this increase did not typically result in appreciably improved OAR sparing. For Eclipse and Corvus, and to a much lesser extent BrainScan, increases in smoothing decreased DC but eventually caused unacceptable losses in plan conformality. Depending on the ITPS, potential benefits from optimizing fluence smoothing levels can be significant, allowing for increases in either efficiency or conformality. Because of variability in smoothing function behavior by ITPS, it is important that users familiarize themselves with the effects of varying smoothing parameters for their respective ITPS. Based on the experience gained here, we provide recommended workflows for each ITPS to best exploit the fluence‐smoothing features of the system.

PACS numbers: 87.56.bd, 87.56.N‐

## I. INTRODUCTION

Intensity‐modulated radiation therapy (IMRT) has evolved to represent a powerful clinical tool and is now widely recognized as the standard of care for a number of disease sites. IMRT is able to produce extremely conformal dose distributions due to its significantly increased degrees of freedom, manifested in the form of large numbers of pencil beams with independently variable intensities. This large number of degrees of freedom requires the use of inverse planning techniques which incorporate computer optimization approaches in the solution of this large scale problem. Due to the increased complexity of the delivered intensity maps, however, this improved conformality may result in potential challenges such as increased delivery time, increased peripheral dose, and technical or mechanical difficulties during treatment delivery. Several methods have been proposed to simplify such treatments through a reduction in complexity, or “smoothing,” of the delivered intensity maps, including beamlet intensity restrictions,^(1)^ direct aperture optimization,^(^ ^2^ ^–^ ^4^ ^)^ and intensity‐modulated beam (IMB) smoothing procedures.^(^ ^5^ ^–^ ^11^ ^)^

Two popular methods of accomplishing fluence map smoothing (also occasionally referred to as *efficiency* improvement) are the application of IMB smoothing filters to decrease the differences in fluence between beamlets,^(5)^ ^,^ ^(6)^ and the incorporation of smoothness terms into the objective function of the optimization algorithm.^(^ ^7^ ^–^ ^11^ ^)^ Both methods seek, ideally, to eliminate any unnecessary and random noise in the fluence maps by decreasing differences in fluence between adjacent beamlets while preserving important features of the overall fluence pattern. Assuming that “unnecessary noise” exists in the fluence map, this can allow for increased delivery efficiency without degradation of plan quality, as decreased fluence complexity requires fewer monitor units for dose delivery.^(12)^ Additionally, smoother intensity maps may also result in dose distributions that are less sensitive to patient motion and treatment alignment uncertainties,^(5)^ ^,^ ^(11)^ and plan quality analyses and treatment execution may be easier.^(7)^ For some treatment planning systems, these benefits may be seen with little to no compromise in plan quality and dose distribution.^(5)^ ^,^ ^(6)^ ^,^ ^(8)^ ^,^ ^(11)^

Commercially available inverse treatment planning systems (ITPS) typically include a “smoothing” or “efficiency” interface. This allows the user to manipulate key parameters of the vendor‐specific smoothing function that control the irregularity or complexity of delivered beam fluence patterns. Most ITPS vendors recommend use of a default set of smoothing parameters within their software. This typically leads to an *average* or *moderate* level of smoothing, or fluence complexity, in order to achieve a desirable balance of complexity and efficiency. It is reasonable to anticipate, however, that a *reduction* of the default smoothing level (i.e. allowing for a more complex fluence map) should result in increased degrees of freedom and, therefore, potential improvement of plan quality for very challenging cases. Alternatively, by increasing the fluence map smoothing from the vendor‐recommended default levels, the user should be able to achieve greater reductions in plan complexity with associated gains in either treatment plan deliverability and/or reduced total body dose. Unfortunately, there exists virtually no peer reviewed literature characterizing the behavior and potential benefits of using such ITPS‐inherent beam smoothing features across multiple planning systems. In this work, we explore the behavior of such smoothing functions in three commercially available ITPSs (Eclipse‐Varian Medical Systems, Palo Alto, CA; BrainScan, BrainLAB AG, Feldkirchen, Germany; and CORVUS, Best Nomos, Pittsburg, PA) for four challenging cases treated by IMRT in our clinic. This analysis does not seek to directly compare one ITPS to another, but rather to improve understanding of each ITPS's smoothing algorithm by describing them in parallel.

## II. MATERIALS AND METHODS

Before an intelligent and systematic evaluation of the impact of smoothing parameters could be performed, it was necessary to acquire a basic understanding of how each planning system accomplished fluence pattern smoothing within its inverse planning process. A brief description for each of the three ITPSs evaluated here follows:

### A. Vendor‐specific smoothing algorithm characteristics

#### A.1 Eclipse

*The following information was obtained from the Varian reference guide for Eclipse algorithms*.^(13)^ For the Eclipse ITPS, fluence smoothing is achieved within the objective function of the ITPS.

A penalty is applied for differences in neighboring fluence values in both the direction of leaf travel (X) and the direction perpendicular to leaf travel (Y). For each beamlet, the fluence value differences between respective neighboring pencil beams are summed together and then multiplied by user‐defined X and Y direction smoothing penalty values, which are then added to the total objective function penalty score. Therefore, these user‐defined values of the X and Y smoothing levels determine the relative priority of these goals in the overall objective function calculation.^(13)^ We observed software‐allowable variables for X and Y to range from 0 to 999. The smoothing component contribution to the overall penalty function increases linearly up to a software‐defined maximum, at which time it becomes a constant value.^(13)^ This mechanism is said to prevent the over‐penalization of large differences in the fluence that are required to achieve dose objectives. The net effect of the fluence smoothing process is to increase the total value of the objective function penalty score for plans with widely varying fluence maps, thereby steering the optimization in a direction leading to smoother fluence maps.

#### A.2 BrainScan

*The following description of BrainScan intensity smoothing functions was obtained from vendor‐provided user documentation* ^(14)^ *and the literature regarding the smoothing algorithm*.^(15)^ For the BrainScan system, smoothing is achieved by way of Bayesian filtering within a dynamically penalized likelihood (DPL) objective function.^(14)^ ^,^ ^(15)^ The objective function penalty increases as the difference in intensity between neighboring beamlets increases, serving to reduce the sharp edges or steep fluence gradients between beamlets. Fluence map smoothing is performed only in the leaf travel direction.^(14)^ The overall smoothness of the BrainScan‐optimized fluence pattern can be adjusted via a user‐defined filter value called “Sharp Edge Smoothing” (SES), which defines the weight of the smoothness term in the DPL algorithm. Vendor‐allowed values for this parameter range from 1% (nearly no smoothing) to 5%, inclusive. The default of 3% is described as a good balance between plan efficiency and conformality. Five percent was chosen by the vendor as a maximum because values above this typically reduce flexibility beyond that required to fulfill plan parameters. BrainScan also includes a function for “Hot Beamlet Restriction” (HBR), which lets the user define the maximum allowed pencil beam monitor units (MU) value “in percent above the MU result for a standard conformal beam treatment.”^(14)^ We observed software‐allowed values for HBR to range from a most restrictive value of 100% (i.e. same MU as a conformal beam) to a least restrictive maximum of 800%, with a vendor‐recommended default value of 400%. The default value is described as “high enough to allow maximum freedom during optimization.”^(14)^

#### A.3 CORVUS

*The following information was obtained through communication with a CORVUS computational physicist*,^(16)^ *as well as the pertinent abstract* ^(17)^ *and patent*.^(18)^ The CORVUS smoothing approach, referred to as *Efficiency* within the CORVUS software, addresses both components of complexity (i.e. monitor units (MU) and segments) independently, but simultaneously, in the objective function.^(^ ^16^ ^–^ ^18^ ^)^ Within this manuscript, Efficiency (capitalized) will refer to this smoothing setting in CORVUS. Monitor units are affected by a vendor‐defined dynamic‐percentage‐range, or “window,” of allowed intensity variability between all pencil beams, and subsequent assignment of a very large penalty for occurrences outside the allowed range. The percentage range value, and the associated penalty, is user‐controlled by the position of the “Efficiency Slider Bar” (range: 0 to 100%, inclusive) located in the prescription section of the software. The effect of the penalty levied for exceeding the allowed percentage range serves, essentially, as a “hard constraint” because of its very large penalty value in the objective function. The total number of delivery segments contained within the optimized plan is controlled by the assessment of a linear penalty that increases as the value of the previously mentioned Efficiency slider is increased. Any number of segments above one is penalized according to the difference between one and the actual number of segments.

### B. Clinical cases and parameters

#### B.1 Treatment sites and their respective prescribed doses & beam arrangements

We evaluated two cases of head and neck cancer, one central nervous system (CNS) case, and one prostate case treated with IMRT in our clinic. Case number one involved a patient with Stage IVB, T4bN0M0 sinonasal carcinoma (SNC), treated with a seven‐field coplanar plan with a 180° couch and collimator rotation (gantry angles: 30°, 80°, 130°, 180°, 230°, 280°, and 330°) to a planning target volume (PTV) dose of 70.2 Gy. The nominal linear accelerator treatment position involves a 180° couch, 180° collimator, and 180° gantry rotation, as defined in the Varian scale. It was difficult to meet the required parameters for the SNC case, as the PTV was located centrally between optic structures on both sides, with the brainstem and spinal cord located posteriorly. Case number two was a patient with a right frontal lobe glioblastoma multiforme (GBM), which involved a five‐field non‐coplanar plan with three different left anterior oblique fields (#1: couch=263°, collimator=180°, and gantry=108°; #2: couch=209°, collimator=299°, and gantry=130°; #3: couch=190°, collimator=94°, and gantry=148°), a right anterior oblique beam (#4: couch=145°, collimator=224°, and gantry=210°), and a right lateral field (#5: couch=180°, collimator=180°, and gantry=270°) with the PTV dose prescribed to 59.4 Gy. The GBM PTV was located in close proximity to the optic chiasm, right optic nerve, and brainstem. Case number three was a patient with Stage IVA, T4aN2bM0 base of tongue squamous cell carcinoma (BOT), treated with a seven‐field coplanar plan with 180° couch and collimator rotation (gantry angles: 0°, 50°, 100°, 150°, 210°, 260°, and 310°) to a PTV dose of 70 Gy. The BOT site required treatment of the neck bilaterally, causing difficulty in sparing typical organs‐at‐risk (OARs) of the head and neck region (e.g. spinal cord, parotid glands, larynx). Case number four was a patient with low‐risk prostate carcinoma (PST), also treated with a seven‐field coplanar plan with 180° degree couch and collimator rotation for each field. Gantry angles were 30°, 80°, 130°, 180°, 230°, 270°, and 320°, with a prescribed PTV dose of 75.6 Gy. The more demanding PST constraints involved the rectum and penile bulb.

#### B.2 Object‐at‐risk (OAR) dose constraints

Challenging OAR dose constraints were required for all plans based on Radiation Therapy Oncology Group (RTOG) protocols, including <1% OAR volume above 54 Gy for the optic nerves and chiasm, and <1% above 50 Gy for the spinal cord. Additional constraints included a maximum dose <54 Gy for the brainstem, maximum doses <45 Gy for the larynx and retina, a mean dose <26 Gy for at least one parotid gland, a mean dose <52.5 Gy to the penile bulb, and 50% of the rectal and bladder volumes receiving over 60 and 65 Gy, respectively.

#### B.3 General inverse treatment planning system (ITPS) specifications

Varian Eclipse v6.5, BrainLAB BrainScan v5.31, and Nomos CORVUS v6.2 were studied for the SNC, GBM, and PST plans. Only Eclipse and CORVUS were studied for BOT planning due to the 10 cm×10 cm field size constraint of the BrainLAB MM3 collimator. The dose calculation grid sizes used for the Eclipse, BrainScan, and CORVUS ITPSs were 2.5 mm, 2 mm, and 2 mm, respectively. Intensity‐modulated beam profiles were converted into leaf motions using DMLC (dynamic multileaf collimation) before calculating the dose‐volume histograms (DVHs). Two types of collimators were evaluated in this study: 1) Varian Millennium 120 leaf MLC (MIL 120) (Varian Medical Systems, Palo Alto, CA) with a pencil beam size of 2.5 mm×5 mm; and 2) BrainLAB MM3 MLC (MM3) (BrainLAB AG, Feldkirchen, Germany) with a pencil beam size of 2 mm×3 mm.

For each ITPS, plans were first optimized using the vendor‐recommended “smoothing” values. Treatment plans were then reoptimized for each subsequent plan on each ITPS, with the only difference being that the user‐definable smoothing parameters were varied for each of the planning systems. To allow fair comparison between optimized plans, all plans were normalized so that 98% of the PTV was covered by 98% of the dose. Normalization values were recorded and graphed for comparison between plans within a given ITPS. For all planning systems, DVHs were constructed for target and critical structures.

#### B.4 Smoothing parameters

##### B.4.1 Eclipse

For the Eclipse ITPS, smoothing was evaluated at (X=0, Y=0; vendor‐defined minimum), (X=20,Y=15), (X=40, Y=30; vendor‐recommended default), (X=60,Y=45), (X=80,Y=60), (X=100,Y=100), (X=150,Y=150), (X=200,Y=200), and (X=999, Y=999; vendor‐defined maximum) for all fields. As mentioned previously, all planning parameters, except smoothing‐related values, were held constant at all times. We note that, while the Eclipse software allows for inverse planning structure dose priorities (or weightings) to be varied between 0 and 1000, it is standard practice in our clinic to limit these values between 0 and 100.

Because both structure‐dose‐priority weights and smoothing (X, Y) penalty weights are embedded components within the Eclipse objective function and, thus, contribute to the total score for a particular plan, we also studied the impact of varying these values relative to each other for Eclipse. We evaluated this effect by keeping the smoothing for all beams constant at the vendor‐recommended default level of (X=40,Y=30), while adjusting all structure‐dose‐priority weights (i.e. for the PTV and OARs) to 1/2,2/3 and 2 times their original values. This was expected to alter the relative importance of the chosen smoothing parameters.

##### B.4.2 BrainScan

For BrainScan, we evaluated SES at 1% (vendor‐defined minimum), 3% (vendor‐recommended default) and 5% (vendor‐defined maximum), with each of these three levels of smoothing run using values of HBR at 100% (vendor‐defined minimum), 400% (vendor‐recommended default), and 800% (vendor‐defined maximum). This resulted in a total of nine combinations of SES and HBR.

##### B.4.3 Corvus

For CORVUS, smoothing was evaluated at 0% (vendor‐defined minimum), 50% (vendor‐recommended starting value), and 100% (vendor‐defined maximum) Efficiency for all plans. Due to large differences in outcome when comparing 0% and 50%, an intermediate value for each site was also evaluated to further characterize CORVUS ITPS behavior within this range.

### C. Delivery complexity (DC)

For the purposes of characterizing the trade‐offs between conformality and delivery efficiency, we defined treatment delivery complexity (DC) as the product of MU and the total number of segments needed *for a particular plan*, divided by the product of the MU and total segments needed *for the default plan* (plan utilizing vendor‐recommended smoothing values). This normalization facilitated comparison between plans at the same disease site for a given ITPS.

For a visual representation of complexity, intensity‐modulated beam (IMB) fluence maps for the minimum, default, and maximum smoothing levels were collected for one of the seven fields of the SNC and PST sites for each ITPS.

### D. Ian Paddick Conformality Index (IPCI)

The Ian Paddick Conformality Index (IPCI), which is the product of a treatment plan's undertreatment and overtreatment ratios, was calculated for each plan using the following equation:

(1) $$IPCI=\frac{{\left({TV}_{PIV}\right)}^{2}}{IV\times PIV)}$$

where $TV_{PIV}$ is the volume of the target covered by the prescription isodose line, *TV* is the total target volume, and *PIV* is the total volume encompassed by the prescription isodose line.

Perfect conformality results in an IPCI of unity, with potential values ranging from 0 to 1.^(19)^ Because we normalized for 98% of the PTV to be covered by 98% of the prescription dose, the maximum IPCI achievable in this analysis is 0.98.

## III. RESULTS

### A. Intensity‐modulated beam (IMB) fluence map comparison

**Figure 1:**
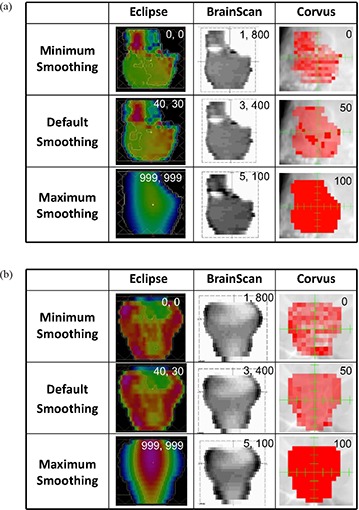
IMB fluence maps for one of seven IMRT fields for: (a) SNC site (gantry angle at 130°), (b) PST site (gantry angle at 180°). Changes in fluence may be seen for each ITPS at minimum, default (vendor‐recommended), and maximum smoothing values. In the upper right of each panel, for Eclipse (X, Y) values are shown, for BrainScan (SES %, HBR %) values are shown, and for CORVUS Efficiency % values are shown.

Figure 1 illustrates how the fluence pattern of an IMB changes with smoothing set to the minimum, default (vendor‐recommended), and maximum smoothing values for the (a) SNC and (b) PST sites. Eclipse can be seen to produce a beam with fluence very uniform in intensity when maximum smoothing values are employed, but only subtle increases in fluence map complexity are noted between the default and minimum smoothing parameters. For BrainScan, variation of fluence complexity is very subtle for all smoothing value changes, although differences are slightly more evident at the 100% HBR level for highly complex sites (SNC).

For CORVUS, the complexity of the fluence pattern decreases relatively consistently as the smoothing (Efficiency) variable is increased, with 100% smoothing resulting in a beam that is essentially uniform in intensity.

### B. Plan normalization

#### B.1 Eclipse

**Figure 2:**
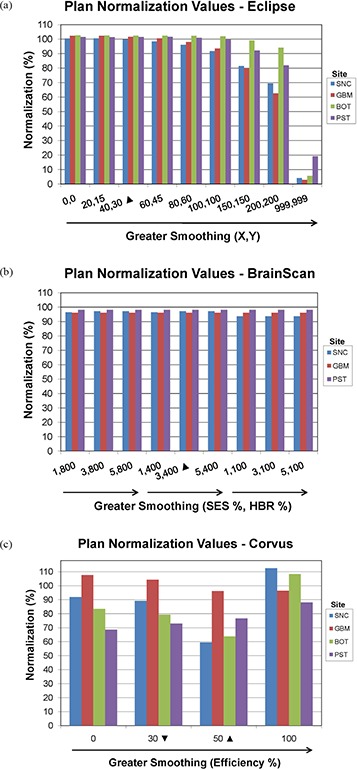
Bar graphs depicting the normalization values required to cover 98% of the PTV by 98% of the dose at various smoothing levels for: (a) Eclipse, (b) BrainScan, (c) CORVUS ITPSs. Vendor‐recommended values are denoted by a solid triangle (▲). The inverted solid triangle (▼) denotes that the CORVUS data shown are for 30% Efficiency, except for BOT which was evaluated at 20%.

Eclipse normalization values were close to 100% of the default smoothing plan for most sites until the smoothing setting approached (X=100,Y=100), after which they were noticeably decreased to achieve desired PTV coverage (Fig. 2(a). Normalization values decreased at a lower smoothing value for the SNC (X=60,Y=45) and GBM (X=80,Y=60) sites. With the maximum smoothing value of (X=999,Y=999), extremely low normalization values ranging from 2.8 to 19% were required.

#### B.2 BrainScan

For BrainScan, normalization values typically remained near 100% and did not differ between plans for the SNC, GBM, and PST sites (Fig. 2(b). One exception is that SNC plans involving 100% HBR, regardless of SES value, required slightly lower normalization values than other plans for that site.

#### B.3 CORVUS

As smoothing increased from 0% to 50% Efficiency, CORVUS' normalization values typically decreased. They then increased at an Efficiency of 100%, although this corresponded with a degradation in OAR sparing (Fig. 2(c).

### C. Dose‐volume histograms (DVHs)

**Figure 3:**
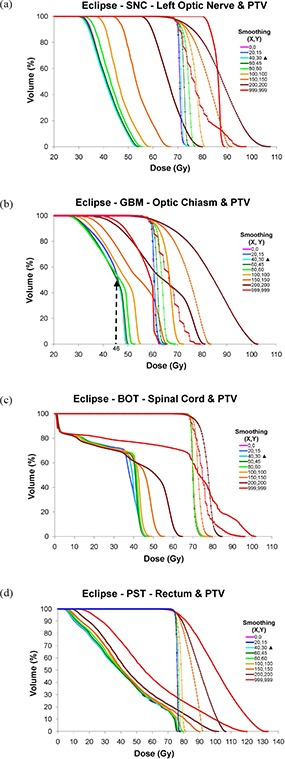
Eclipse DVHs for: (a) SNC left optic nerve and PTV, (b) GBM optic chiasm and PTV, (c) BOT spinal cord and PTV, (d) PST rectum and PTV. The first number of the legend is the X value; the second is the Y value. OAR series are shown as thick solid lines, and PTV series are depicted by thin diamond‐ticked lines. Vendor‐recommended smoothing occurs at (X=40,Y=30), denoted by a solid triangle (▲). The minimum allowable smoothing value is 0, and the maximum is 999.

Figures 3, 5 and 7 show DVHs for the PTV and OARs of most concern for exceeding dose tolerance for each disease site planned on the Eclipse, BrainScan and CORVUS ITPSs, respectively. The OARs are the left optic nerve for the SNC site, the optic chiasm for the GBM site, the spinal cord for the BOT site, and the rectum for the PST site. Recall that for all plans in this study, target coverage was normalized to be equivalent with 98% of the PTV receiving 98% of the prescribed dose. Please refer to the normalization values depicted in Fig. 2 to provide context to the following DVH descriptions.

**Figure 4:**
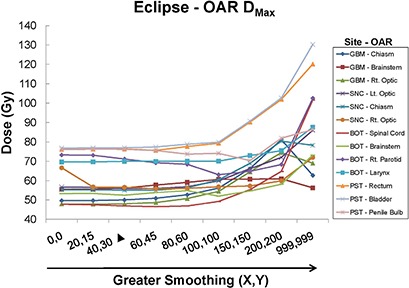
Eclipse OAR maximum doses $\left(D_{\max}\right)$ for all sites as a function of plan smoothing. Vendor‐recommended smoothing occurs at (X=40,Y=30), denoted by a solid triangle (▲).

**Figure 5:**
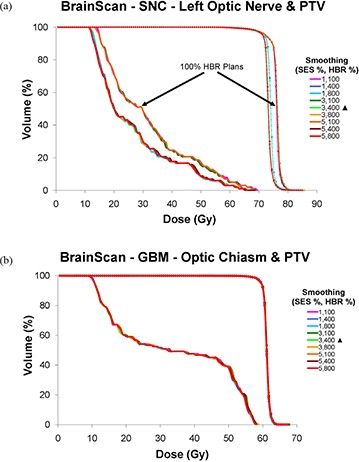
BrainScan DVHs for: (a) SNC left optic nerve and PTV, (b) GBM optic chiasm and PTV. The first number of the legend indicates the % SES, and the second refers to the % HBR value. SES values include 1% (minimum), 3% (vendor‐recommended), and 5% (maximum), while HBR values include 100% (minimum), 400% (vendor‐recommended), and 800% (maximum). OAR series are shown as thick solid lines, and PTV series are depicted by thin diamond‐ticked lines. Vendor‐recommended values are denoted by a solid triangle (▲).

#### C.1 Eclipse

##### C.1.1 Eclipse – SNC site

Figure 3(a) depicts virtually identical left optic nerve DVH curves when smoothing parameters ranging from (X=0,Y=0) to (X=60,Y=45) were employed. Once a smoothing level of (X=80,Y=60) was reached, a steady increase in dose delivered to the left optic nerve occurred. The highest optic nerve dose can be seen at the maximum smoothing level of (X=999,Y=999). As normalization values were lowered, PTV maximum doses increased, as expected. This resulted in very high maximum plan doses up to 157% the prescribed dose, with the required MU increased by up to six times the default plan.

##### C.1.2 Eclipse – GBM site

Figure 3(b) shows that once the cumulative dose to the optic chiasm reached a value of approximately 46 Gy (indicated by dashed arrow), dose to the chiasm increased gradually as smoothing values were increased, as might be naturally anticipated. Below 46 Gy, dose to the chiasm as a function of smoothing was not as intuitive in behavior in that some plans run with lower relative smoothing values actually resulted in greater dose delivered (i.e. for (X=0,Y=0) to (X=80,Y=60)). For the highly smoothed (X=999,Y=999) plan, an extremely low normalization value of 2.9% was required to achieve PTV dose coverage. This resulted in unpredictable behavior of structure DVHs, especially beyond 54 Gy. As with the SNC site, the lower normalization values associated with increased smoothing increased the maximum PTV doses. The maximum plan dose reached a high of 172%, with the required MU increased by up to eight times the default plan.

##### C.1.3 Eclipse – BOT site

Figure 3(c) depicts DVH data for the most challenging structure in the BOT site – the spinal cord. In general, dose to the spinal cord increased progressively as smoothing was increased, with the maximum dose converging to a constant value of approximately 47 Gy for smoothing values of (X=80,Y=60) and lower. Above smoothing levels of (X=80,Y=60), the maximum dose to the spinal cord can be seen to increase dramatically. As normalization values were lowered, PTV maximum doses were increased.

##### C.1.4 Eclipse – PST site

Figure 3(d) depicts behavior of PST plans at various smoothing values. Consistent with the other sites, plan degradation begins to occur above smoothing values of (X=60,Y=45).

##### C.1.5 Eclipse – all sites

Figure 4 presents maximum OAR dose (Dmax) as a surrogate for OAR DVH data for the various sites. Consistent with the results seen for OARs in Fig. 3, sparing typically decreases beyond smoothing values of (X=40,Y=30) to (X=60,Y=45), with all OARs having a higher $D_{\max}$ by (X=200,Y=200). Of note, the $D_{\max}$ for some OARs is at a minimum around default smoothing values.

#### C.2 BrainScan

##### C.2.1 BrainScan – SNC site

The DVH curves of the very demanding SNC plan (Fig. 5(a) appear to have stratified according to HBR value (i.e. either “enough freedom” at 400% or greater, or “not enough” freedom at 100%). This phenomenon was most pronounced for the left optic nerve, likely because its tolerance was pushed the hardest of any OAR due to its proximity to the PTV. However, this finding was also present for all other OARs for the SNC site. The quality of the plan, as characterized by sparing of the most difficult OAR (i.e. left optic nerve), was essentially unaffected by the SES value within the two stratified HBR groups. Any SNC plan utilizing 100% HBR (labeled pink, green and orange), regardless of SES value, required lower normalization values, resulting in larger DVH shoulders and higher PTV maximum doses.

##### C.2.2 BrainScan – GBM and PST sites

Figure 5(b) shows that for the BrainScan GBM site, regardless of HBR and SES value combinations, the optic chiasm DVHs were virtually identical. Contrary to the SNC site, all HBR and SES value combinations performed nearly identically for GBM PTV coverage. Therefore, normalization values were equal for all plans, with PTV DVHs overlaying each other. As with the GBM site, the PST OAR and PTV DVHs were essentially unchanged for the various HBR and SES values (graph not shown).

##### C.2.3 BrainScan – all sites

**Figure 6:**
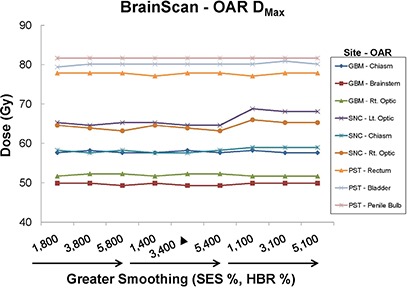
BrainScan OAR maximum doses $\left(D_{\max}\right)$ for all sites as a function of plan smoothing. Vendor‐recommended values of 3% SES and 400% HBR are denoted by a solid triangle (▲).

Figure 6 depicts $D_{\max}$ for BrainScan OARs of concern for the SNC, GBM, and PST sites. Consistent with findings described for BrainScan thus far, $D_{\max}$ remained near constant across all smoothing values for the GBM and PST sites. For the more challenging SNC site, OAR $D_{\max}$ was highest for the 100% HBR plans.

#### C.3 CORVUS

##### C.3.1 CORVUS – SNC, GBM, BOT and PST sites

**Figure 7:**
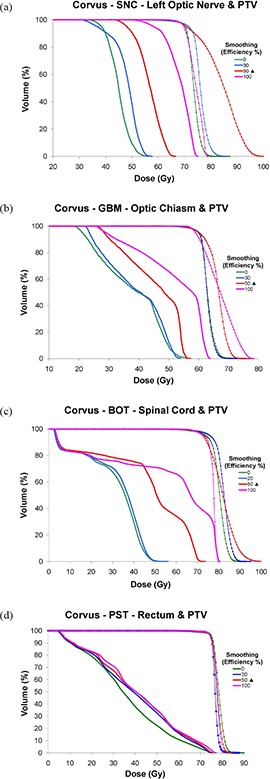
CORVUS DVHs for: (a) SNC left optic nerve and PTV, (b) GBM chiasm and PTV, (c) BOT spinal cord and PTV, (d) PST rectum and PTV evaluated at various % Efficiency levels. OAR series are shown as thick solid lines, and PTV series are depicted by thin diamond‐ticked lines. Vendor‐recommended smoothing occurs at 50%, denoted by a solid triangle (▲). The minimum allowable smoothing is 0%, and the maximum 100%.

Figures 7(a))–7(d) show that increasing smoothing resulted in a progressive increase in total dose to the OAR for each site, as expected. The closer a particular OAR structure was to the PTV, the more challenging it was to spare and, thus, the greater the change in its received dose when smoothing was varied. PTV DVH shoulders generally increased from 0% to 50% Efficiency for the various sites, as expected. Although the shoulder for 100% was sharper than for 50% with a higher corresponding normalization value for the SNC and BOT sites (Figs. 7(a) and (c)), this appeared to have come at the expense of OAR sparing.

##### C.3.2 CORVUS – all sites

**Figure 8:**
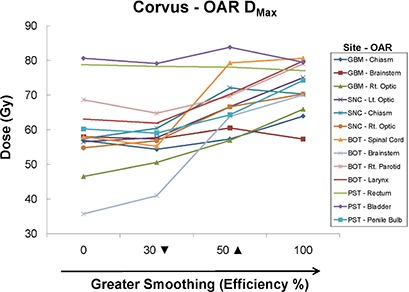
CORVUS OAR maximum doses $\left(D_{\max}\right)$ for all sites as a function of plan smoothing. Default smoothing occurs at 50% Efficiency, denoted by a solid triangle (▲). The inverted triangle (▼) denotes that the data shown are for 30% Efficiency, except for BOT which was evaluated at 20%.

In general, $D_{\max}$ increased as Efficiency increased for all OARs (Fig. 8), with a minimum achieved at a smoothing value under 50% Efficiency.

### D. Impact of varying smoothing values relative to structure‐dose‐priority‐weights for Eclipse organ‐at‐risk (OAR) dose‐volume‐histograms (DVHs)

As mentioned in the Materials and Methods (Section II), smoothing values and structure‐dose‐priority weights within the Eclipse ITPS are both user‐defined contributors to the overall objective function score. Therefore, by varying one set of parameters relative to the other, the user can change the ratio of the two penalty components to each other and, therefore, the relative contribution of the smoothing penalty versus the structure‐dose penalty. This is important to realize for the Eclipse system because any characterization of the behavior of various smoothing levels must, therefore, be associated with the range of structure‐dose‐priority weights used for that plan. In this section, we present the findings of work we performed to verify our expectation that our results would depend on the ratio of the two sets of values to each other.

**Figure 9:**
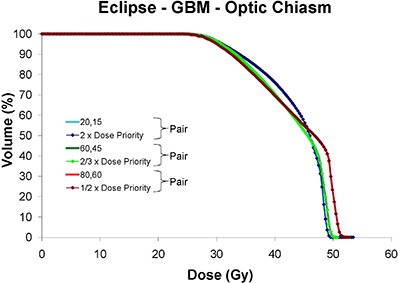
Eclipse optic chiasm DVHs for the GBM site with the ratio of the smoothing value to the structure‐dose‐priority weight kept constant for various pairs of plans. These pairs, as noted in brackets, include (X=20,Y=15) and (2×Dose Priority), (X=60,Y=45) and (2/3×Dose Priority), and (X=80,Y=60) and (1/2×Dose Priority).

Figure 9 shows, for the GBM site planned on the Eclipse ITPS, the DVH data for the chiasm at various smoothing levels as well as when smoothing levels were held constant at the default level (i.e. X=40,Y=30) and all structure‐dose‐priority weights were scaled either up or down uniformly. The DVH data demonstrates that optimized DVH results remain similar if either smoothing or dose‐priority changes are made that keep the ratios between the two sets of constraints similar. The same conclusions could be drawn from similar graphs of the SNC, BOT and PST sites, with the DVHs of plan “pairs” having equivalent ratios nearly superimposed on one another in all cases.

### E. Delivery complexity (DC) and Ian Paddick Conformality Index (IPCI) as a function of variable smoothing

#### E.1 Eclipse

**Figure 10:**
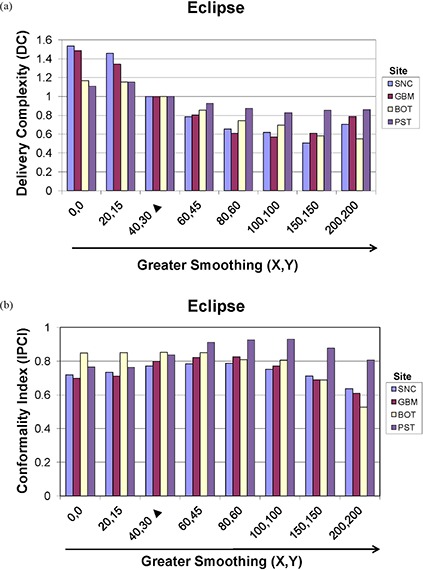
Eclipse (a) delivery complexity (DC) and (b) Ian Paddick Conformality Index (IPCI) values for the SNC, GBM, BOT and PST sites planned using (X, Y) smoothing values ranging from 0 to 200 (higher smoothing values not shown to avoid skewing scale of presented data). Vendor‐recommended values are denoted by a solid triangle (▲).

DC and IPCI for the Eclipse ITPS are evaluated in Figs. 10(a) and 10(b), respectively. Figure 10(a) shows, in general, that as Eclipse smoothing values are increased, DC decreases, with the normalized DC value of unity seen at the vendor‐recommended default smoothing level (i.e. X=40,Y=30). An interesting exception to this behavior was observed as the DC curve turned upward again at very high values of smoothing. At smoothing values of (X=999,Y=999), plan normalization values were extremely low, ranging from 2.9 to 19%, and the DC rose to a value almost eight times that of the default plan for all sites. Figure 10(b) shows that the IPCI achieves a maximum value for all four treatment sites at or slightly above vendor‐recommended default smoothing values and then, expectedly, begins to decrease as smoothing is increased. The fact that smoothing values lower than the default values did not result in improved conformality was unexpected.

#### E.2 BrainScan

**Figure 11:**
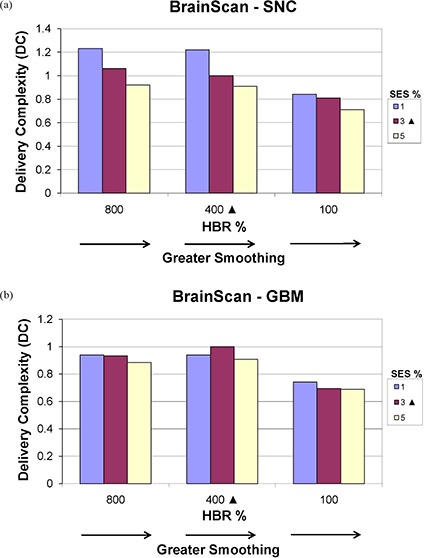
BrainScan delivery complexity (DC) values for the (a) SNC and (b) GBM sites, planned using SES values of 1% (minimum), 3% (vendor‐recommended), and 5% (maximum) each at HBR levels of 100% (minimum), 400% (vendor‐recommended), and 800% (maximum). Vendor‐recommended values are denoted by a solid triangle (▲).

**Figure 12:**
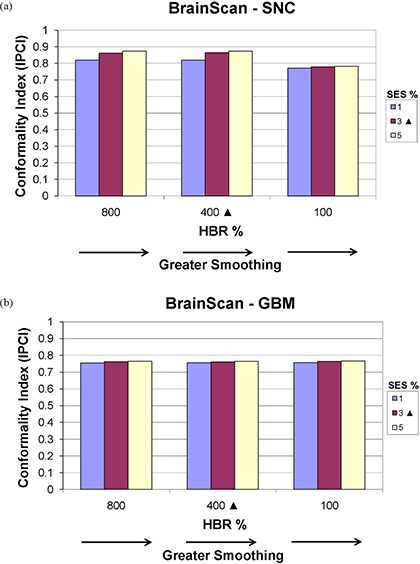
BrainScan Ian Paddick Conformality Index (IPCI) values for the (a) SNC and (b) GBM sites, planned using SES values of 1% (minimum), 3% (vendor‐recommended), and 5% (maximum) each at HBR levels of 100% (minimum), 400% (vendor‐recommended), and 800% (maximum). Vendor‐recommended values are denoted by a solid triangle (▲).

Figures 11 and 12 show how DC and IPCI, respectively, are affected by altering SES and HBR for BrainScan. Figure 11 shows that as smoothing (SES) increases DC generally decreases, as expected, for a given HBR value, although this is most apparent for the SNC plan (Fig. 11(a). While no clear decrease in DC is noted between 800% and 400% HBR for both the SNC and GBM sites, a further reduction to 100% resulted in decreased DC values. Unexpectedly, Fig. 12 shows a subtle increase in IPCI with increased SES values, with no effect on OAR dosing (refer to Fig. 5). Although Fig. 12(a) shows decreased IPCI for the SNC site with 100% HBR as would be expected, this effect is not seen for the GBM (Figure 12(b) or PST (not shown) sites.

#### E.3 CORVUS

**Figure 13:**
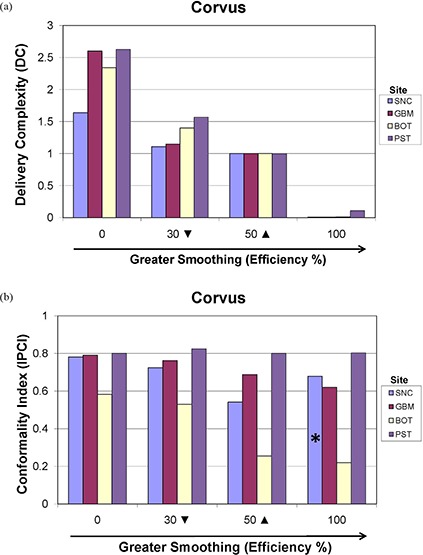
CORVUS (a) delivery complexity (DC) and (b) Ian Paddick Complexity Index (IPCI) values for the SNC, GBM, BOT and PST sites evaluated at various Efficiency levels. Default smoothing occurs at 50%, denoted by a solid triangle (▲). The minimum allowable smoothing is 0%, and the maximum 100%. The asterisk denotes a finding of increased IPCI for the SNC site at 100% over 50%, which was achieved because of OAR overdosing. The inverted triangle (▼) denotes that the data shown are for 30% Efficiency, except for BOT which was evaluated at 20%.

For the CORVUS ITPS, Figs. 13(a) and 13(b) generally indicate that as Efficiency (smoothing) increases, both DC and IPCI decrease, as might be expected. However, an interesting exception is that the IPCI is seen to be higher for the SNC 100% Efficiency plan than for the 50% Efficiency plan (see Fig. 13(b) ‐ asterisk on bar). This behavior can be better understood by recognizing that this improvement in conformality was achieved at the expense of the OARs, which were significantly overdosed for the 100% plan (see Fig. 7(a).

### F. Impact of smoothing to structure‐dose‐priority weight ratio on Eclipse DC and IPCI

**Figure 14:**
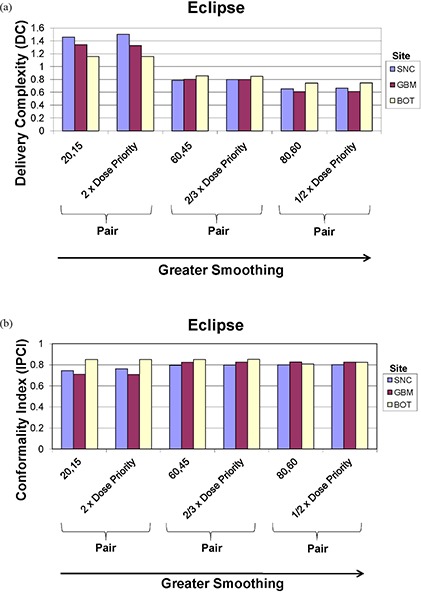
Eclipse (a) delivery complexity (DC) and (b) Ian Paddick Conformality Index (IPCI) for the SNC, GBM, and BOT sites, comparing pairs of plans when the ratio of the smoothing value to the structure‐dose‐priority weight is kept constant. The pairs evaluated (noted in brackets) include (X=20,Y=15) and (2×Dose Priority), (X=60,Y=45) and (2/3×Dose Priority), and (X=80,Y=60) and (1/2×Dose Priority). PST data not shown to avoid unnecessary graph complexity, as results are similar to all sites currently depicted.

Figures 14(a) and 14(b) show that if either smoothing or structure‐dose‐priority weights are altered so that the ratio of their user‐defined values remains the same, the results are nearly identical DC and IPCI values between the pairs.

### G. Smoothing parameter effect on isodose distributions

#### G.1 Eclipse

**Figure 15:**
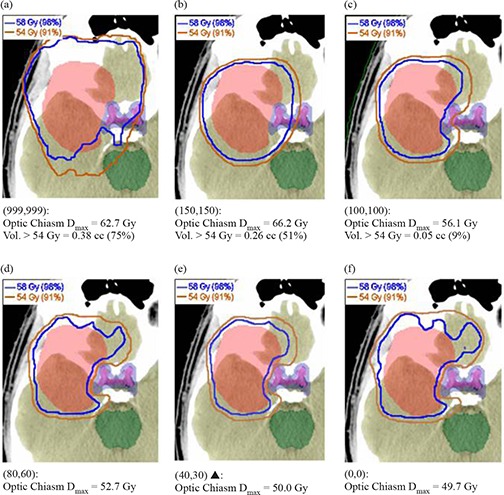
Eclipse GBM isodose lines at the axial slice where left optic nerve sparing is most difficult. The blue line is the 98% (58.2 Gy) isodose line, which is the prescription dose with which we planned to cover 98% of the PTV (red). The orange line is the 91% (54 Gy) isodose line, which is the dose tolerance for the optic nerves and chiasm. Of particular concern is the chiasm, shown in magenta, surrounded by a 3 mm safety margin in blue. For each smoothing value in panels (a) through (f), the maximum dose to the chiasm $\left(D_{\max}\right)$ is reported, as is the volume of the chiasm >54 Gy in both cubic centimeters (cc) and in percent of the total chiasm volume (where appropriate). Vendor‐recommended values of (X=40,Y=30) are denoted by a solid triangle (▲).

Figure 15 shows that as (X, Y) smoothing values are decreased, Eclipse conformality increases and OAR dose decreases. Of particular importance is the optic chiasm, which can be seen to exceed dose tolerance at and above (X=100,Y=100). Significant conformality degradation occurs when (X≥150,Y≥150), where the ITPS has apparently lost the freedom required to create concave dose distributions.

#### G.2 BrianScan

**Figure 16:**
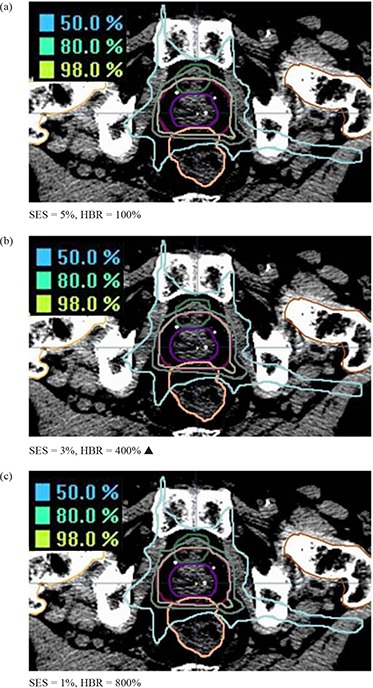
BrainScan PST (GTV in purple & PTV in maroon) isodose lines at an axial slice showing both the rectum (pink) and bladder (green) at the maximum (SES=5%,HBR=100%), vendor recommended (SES=3%,HBR=400%), and minimum (SES=1%,HBR=800%) smoothing values. OAR sparing does not appreciably differ between smoothing values. Vendor‐recommended values are denoted by a solid triangle (▲).

BrianScan dosimetry varies little across the allowable user‐defined range of smoothing values, as shown by a representative axial slice of the PST site (Fig. 16) where differences are near imperceptible between plans.

#### G.3 CORVUS

**Figure 17:**
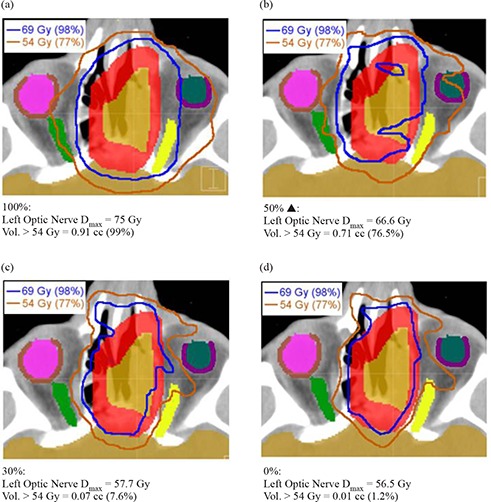
CORVUS SNC isodose lines at the axial slice where left optic nerve sparing is most difficult. The blue line is the 98% (69 Gy) isodose line, which is the prescription dose with which we planned to cover 98% of the PTV (red). The orange line is the 77% (54 Gy) isodose line, which is the dose tolerance for the optic nerves (left in yellow; right in green) and chiasm (not shown). Of particular concern is the left optic nerve. For each Efficiency value in panels (a) through (d), the maximum dose to the left optic nerve $\left(D_{\max}\right)$ is reported, as is the volume of the left optic nerve >54 Gy in both cubic centimeters (cc) and in percent of the total left optic nerve volume. The vendor‐recommended value of 50% is denoted by a solid triangle (▲).

Figure 17 shows that decreasing CORVUS smoothing below 50% may be essential to meet plan goals. For the SNC site, a plan with an acceptable dose received by the left optic nerve is possible at an Efficiency value of 30%.

## IV. DISCUSSION

This work quantifies how variable the application of vendor‐supplied, fluence‐smoothing approaches can be on the resulting optimized treatment plan, depending on the specific ITPS used. This is a fact that may well be under‐appreciated by many users. It seems reasonable to assume that for each respective patient there may be a critical complexity level needed to achieve a satisfactory plan, beyond which extra complexity does not appreciably improve plan quality but, rather, produces plans that take significantly longer to deliver with little or no plan improvement.^(8)^ The potential benefits of smoothing are clear, in that the possibility exists to match the complexity needed to that allowed, with associated gains in efficiency and plan conformality. However, it is important that achievement of this critical complexity level not be prevented or limited by “over‐smoothing.” This concern is particularly relevant in challenging areas such as the head and neck, CNS, and pelvis, where numerous critical structures are in close proximity to target volumes. Appropriate use of the fluence map smoothing functionality inherent to typical IMRT ITPSs can potentially result in valuable improvements in plan quality, such as reduced critical structure maximum doses. This benefit is seen with CORVUS, where decreases in smoothing resulted in a higher level of conformality and complexity, which was often necessary to achieve plan specifications. Conversely, and again according to expectations, increased smoothing values resulted in decreased conformality and complexity. For Eclipse, the effects of changing smoothing values were more difficult to predict, sometimes leading to expected trends and other times leading to unanticipated results. For the BrainScan ITPS, differences in plan quality were very subtle, irrespective of the smoothing levels used.

### A. Planning system summary

#### A.1 Eclipse

Changes in conformality secondary to smoothing level variation are less predictable for Eclipse than the other ITPSs, although such changes do not appear necessary to achieve an acceptable plan in most cases. The vendor‐recommended values of (X=40,Y=30) were found to be suitable by the institutions that did the initial beta testing of IMRT optimization with Eclipse.^(20)^ Mayo and Urie,^(21)^ as described in their user manual for Eclipse, evaluated “no smoothing” (X=0,Y=0), “moderate smoothing” (X=40,Y=30), and “heavy smoothing” (X=90,Y=90) for a head and neck plan phantom. Although the actual structure‐dose‐priority weights used were not specified for this particular analysis, the authors recommended using between 0 and 100 because they historically used this range in an older ITPS (CADPlan). This recommendation was not reported to be based on any empirical testing of values outside that range. The plan with “no smoothing” resulted in good target coverage, but Mayo and Urie noted “islands of hot spots.” They reported DVH analyses of the PTV and the spinal cord, showing significant degradation of PTV coverage and spinal cord sparing with “heavy smoothing.” Although structure DVHs for the default and “no smoothing” plans were reported to be nearly identical, the “no smoothing” plan was found to require 180% more monitor units to deliver. Mayo and Urie stated that the 20% decrease in monitor units found with the “heavy smoothing” plan did not justify the decrease in plan quality. Therefore, they concluded that using “moderate smoothing values” near the default level was a “good compromise” between conformality and required MU in most clinical cases, and they suggested keeping smoothing values between 0 and 100.^21^

The methods used in our study contrast with those of Mayo and Urie in that we normalized for 98% PTV coverage by 98% of the dose for all plans. Therefore, all of the Eclipse plans studied here were required to have similar PTV coverage, with subsequent differences in DC and IPCI values and OAR sparing. In our experience, the smoothing levels which tended to result in the lowest doses to OARs generally occurred at or near vendor‐recommended values (i.e. X=20 to 80, Y=15 to 60). Outside this range, there was less OAR sparing and, in general, the default smoothing plan performed the best (i.e. X=40, Y=30). Worse plan quality with lower smoothing values is contrary to our expectations, as we believed that increased degrees of freedom would allow better conformality. Despite this expectation, plans run with little to no smoothing demonstrated increases in DC with no clear increase in plan conformality or OAR sparing. Our observation of a consistent decrease in IPCI and OAR sparing at (X, Y) smoothing levels ≥100 was expected, due to a loss of beam complexity. The observed IPCI values reached a maximum slightly above the vendor‐recommended smoothing values, contrary to our expectations of a maximum at minimum smoothing. Figures 3(a))–3(d) show a progressive increase in the PTV DVH shoulder starting from (X=0,Y=0). This indicates that as smoothing values were increased, PTV coverage decreased, thus requiring increasingly lower normalization values thereby causing increased over‐treatment of surrounding tissues. This also resulted in higher DC values second to the MU increase. Figure 1 shows little decrease in beam complexity between the minimum and vendor‐recommended default smoothing values, but as (X, Y) values were increased further, the fluence across the IMB became more constant, with an almost completely smooth beam observed with maximal (X, Y) values.

We also sought to characterize the relationship between smoothing values and structure‐dose‐priority weights. The pairs investigated in this report included (X=20,Y=15) and (2×dose priority values), (X=60,Y=45) and (2/3×dose priority values), and (X=80,Y=60) and (1/2×dose priority values). Figure 9 depicts nearly identical OAR DVH curves for the pairs, and Fig. 14 shows matching DC and IPCI values. This point is important, as it demonstrates that dose priority levels should be kept between 0 and 100 for our results to remain relevant.

Since plan quality may start to diminish for Eclipse if smoothing values are altered much from the vendor‐recommended values of (X=40,Y=30), we suggest starting the planning process by keeping structure‐dose‐priority weights between 0 and 100 and leaving (X, Y) smoothing at the vendor‐recommended values. After a plan is optimized using these parameters, we then recommend varying the smoothing levels within the ranges of (X=40 to 80) and (Y=30 to 60) to identify any potential benefits. In our experience, use of values below this level will likely increase complexity with a low likelihood of increased conformality, while values in this recommended range may decrease complexity with potential improvements in conformality.

#### A.2 BrainScan

For the disease sites studied here, we observed that altering BrainScan's smoothing values (i.e. SES) had almost no impact on OAR DVHs, indicating little relative importance of this parameter within the allowed user‐defined value range in the ITPS's objective function. Fluence map complexity decreased as smoothing was increased, as would be expected. However, the magnitude of this effect was very small, as seen with the SNC and PST sites in Fig. 1. Unanticipated behavior sometimes occurred, such as when the IPCI increased with increased smoothing (Fig. 12).

According to the BrainScan documentation, HBR is not relevant for standard cases. The default value of 400% is said to be set high enough to enable maximum freedom for the optimization,^(14)^ although no supporting data is offered. This claim is upheld, however, by our results, which showed that increasing values of HBR beyond the default to 800% caused near negligible increases in plan DC, with no apparent improvement in IPCI. The BrainScan user manual warns that extremely low values may be too restrictive, thus creating an undesirable plan.^(14)^ Our results support this statement as well; for the SNC site, the minimum allowable HBR value of 100% resulted in significant degradation in OAR sparing (Fig. 5(a). Increased challenge with PTV coverage can be noted as well, as the 100% HBR plans had broader shoulders for the SNC PTV DVHs, reflecting lower normalization values were required for these settings (Fig. 2(b). In turn, this increase in MU resulted in an increased DC. It appears reasonable to attribute this to the HBR value being too limiting to achieve adequate OAR sparing and PTV coverage. However, for less complicated sites such as GBM and PST, a 100% HBR value allowed for decreased DC with no clear detriments in plan conformality. No changes in normalization values were required to achieve desired PTV coverage for the GBM or PST sites for any SES or HBR setting.

Based on these results for the BrainScan ITPS, we recommend starting the planning process using vendor‐recommended default SES and HBR parameters. As expected, increasing SES decreased DC values, but this also counterintuitively increased the IPCI slightly. Therefore, after achieving an acceptable plan, it may be worthwhile to see if increasing SES towards 5% allows for decreased DC without conformality degradation. With regards to HBR, decreasing the default value of 400% to 100% may decrease plan complexity. However, for more complicated sites (e.g. SNC), one must ensure this does not come at the cost of worsened conformality.

#### A.3 CORVUS

In general, CORVUS behaved as expected, showing improved plan conformality with decreased smoothing (i.e. decreased Efficiency). Specifically, as smoothing was decreased, increased OAR sparing and a higher IPCI were achieved. This came at the expense of increased MU and segments and, therefore, an increased DC factor. The ability to achieve increased OAR sparing with increased complexity is not surprising, given how much the complexity of CORVUS IMBs can vary with Efficiency setting, as seen in Fig. 1. The default 50% Efficiency level often seemed to be at the cusp of allowing the necessary degrees of freedom to achieve all plan goals. In the sites we evaluated, this value was not always sufficient to allow for both conformal target coverage and OAR sparing, although the system endeavored to do both.

Therefore, we ran additional CORVUS plans at Efficiency levels between 0 and 50% to determine the maximum Efficiency value that allowed satisfaction of all plan goals. For the GBM site, it was necessary to decrease the Efficiency level to 30% before we could both cover the PTV and keep <1% of the optic chiasm volume above 54 Gy. The maximum Efficiency for the BOT case that still kept <1% of the spinal cord volume above 50 Gy while maintaining PTV coverage was 20%. To further illustrate the behavior of CORVUS at its “tipping point” of allowing an adequate amount of freedom, for the SNC plan we collected axial images displaying both the 98% (69 Gy, denoted in blue) and 77% (54 Gy, denoted in orange) isodose lines (Fig.17). These values represented our PTV (shown in red) coverage goal and our dose constraint for the optic nerves/chiasm, respectively. Figure 7(a) shows that the PTV was covered well by the 98% isodose line at 100% Efficiency, but Figure 17(a) elucidates why this was possible – virtually the entire left optic nerve (yellow) was receiving over the 54 Gy limit. In Fig. 17(b), the default Efficiency value of 50% is high enough to allow only very minor sparing of the left optic nerve, and this comes at the expense of undertreatment of part of the PTV and overtreatment of significant amounts of surrounding normal tissue, particularly to the right side of the PTV. This resulted in the worst IPCI of all the plans evaluated (Fig. 13(b), likely due to the ITPS struggling to balance compromises of both PTV coverage and OAR sparing. Because this plan had the worst undertreatment ratio, the lowest normalization value (Fig. 2(c) was required among the plans evaluated to achieve the required PTV coverage. This explains why the 50% Efficiency plan has the highest PTV dose, as seen in Fig. 7(a). This effect on PTV dose is also present for the BOT site (Fig. 7(c), and to a lesser extent the GBM site (Fig. 7(b)).

Figure 17(c) illustrates that if CORVUS Efficiency is decreased further to 30%, there are enough degrees of freedom to spare most of the left optic nerve, as well as conform to the PTV. The DC here is only 11% higher than at the default Efficiency of 50%. A further decrease in Efficiency to 0% (Fig. 17(d) allowed additional improvements in IPCI (Fig. 13(b) and OAR sparing (Fig. 7(a), but at significant cost to efficiency, with a DC value 164% of the default plan (Fig. 13(a).

Based on the data collected here, our recommendation regarding the use of the Efficiency parameter for CORVUS is that if difficulty is encountered in meeting desired dosimetric goals, decreasing the Efficiency value is a reasonable option to potentially improve PTV coverage and OAR sparing. However, it is important to review the plan carefully for resulting increases in complexity (i.e. MU and segments), as these may be significant.

### B. Study Limitation

If PTV coverage is “significantly” inadequate, standard practice at our institution is to reoptimize the treatment plan with altered structure‐dose‐priority weights, versus simply renormalizing, as we did in this study. While some clinics may only renormalize to achieve the required target dose specifications, this technique will most likely increase the dose not just to the PTV but to all structures, including OARs. For cases where the PTV is underdosed by more than a few percent, we believe it is prudent to reoptimize with newly defined structure‐dose‐priority weights. Unfortunately, reoptimizing with altered structure‐dose‐priority weights for each plan would have limited our ability to compare results between plans. We believe that normalizing for identical target coverage was necessary in this study to allow accurate and meaningful plan comparisons.

## V. CONCLUSIONS

The behavior of fluence‐map smoothing functions varies widely by vendor. Depending on the inverse treatment planning system used, the potential benefits of optimizing fluence smoothing levels can be significant, allowing for increases in either delivery efficiency or plan conformality. Our observations for the clinical cases explored here using the Eclipse, BrainScan and CORVUS ITPSs can hopefully serve as a starting guideline regarding what can be expected when smoothing/efficiency is altered within each of these specific planning systems (please see Appendix for summary of recommendations). Because of the variability in smoothing function behavior by ITPS vendor, it is important that users familiarize themselves with the effects of varying smoothing/efficiency parameters for their respective ITPSs.

## Supporting information

Supplementary Material

## Eclipse v6.5

- Optimize plan first with vendor‐recommended default smoothing parameters (X=40,Y=30).
- Keep structure‐dose‐priority weights between 0 and 100.
- Consider increasing smoothing levels, keeping X≤80 and Y≤60, with the intended benefit of decreasing complexity without compromising PTV coverage or OAR sparing.
- It is imperative that all OARs are checked carefully for clinically relevant increases in dose.
- No clear or consistent benefit from decreasing smoothing was observed in the cases we evaluated.

## BrainScan v5.31

- Optimize plan first with vendor‐recommended default parameters of SES=3% and HBR=400%.
- Determine if increased SES and decreased HBR values allow decreased complexity without detriment to conformality for your particular case.
- Decreasing SES and increasing HBR values do not appear useful for the cases studied here, as these changes increased complexity with no apparent conformality gains.

## CORVUS v6.2

- Optimize plan first using the vendor‐recommended default 50% Efficiency setting.
- If one is finding difficulty meeting a plan's dosimetric goals, decreasing the Efficiency parameter is a reliable way to increase PTV conformality and OAR sparing.
- However, one must ensure dosimetric improvements are worth the cost of increased plan complexity.
