# Supplementary material for: Evaluation of fluence‐smoothing feature for three IMRT planning systems
Source: J Appl Clin Med Phys. 2010 Apr 16;11(2):33–61. doi: 10.1120/jacmp.v11i2.3035 (PMC5719958; doi:10.1120/jacmp.v11i2.3035)
Supplement: Supplementary file 1 — Supplementary Material [file ACM2-11-033-s001.doc]

*Appendix - Summary of Recommendations:*

*Eclipse*

- Optimize plan first with vendor-recommended default smoothing parameters (X=40, Y=30).
- Keep structure-dose-priority-weights between 0 and 100.
- Consider increasing smoothing levels, keeping X≤80 and Y≤60, with the intended benefit of decreasing complexity without compromising PTV coverage or OAR sparing.
- It is imperative that all OAR’s are checked carefully for clinically relevant increases in dose.
- No clear or consistent benefit from decreasing smoothing was observed in the cases we evaluated.

*BrainScan*

- Optimize plan first with vendor-recommended default parameters of SES = 3% and HBR = 400%.
- Determine if increased SES and decreased HBR values allow decreased complexity without detriment to conformality for your particular case.
- Decreasing SES and increasing HBR values do not appear useful for the cases studied here, as these changes increased complexity with no apparent conformality gains.

*Corvus*

- Optimize plan first using the vendor-recommended default 50% Efficiency setting.
- If one is finding difficulty meeting a plan’s dosimetric goals, decreasing the Efficiency parameter is a reliable way to increase PTV conformality and OAR sparing.
- However, one must ensure dosimetric improvements are worth the cost of increased plan complexity.
